# Enlarged cross-sectional area of the left vagus nerve in patients with major depressive disorder

**DOI:** 10.3389/fpsyt.2023.1237983

**Published:** 2023-07-31

**Authors:** Lisa Sofie Schreiber, David Wozniak, Erik Scheller, Elise Böttcher, Johann Otto Pelz, Frank M. Schmidt

**Affiliations:** ^1^Department of Psychiatry and Psychotherapy, Leipzig University Hospital, Leipzig, Germany; ^2^Department of Neurology, Leipzig University Hospital, Leipzig, Germany

**Keywords:** major depressive disorder, vagus nerve, ultrasound, autonomic nervous system, gut-brain axis, inflammation, depression

## Abstract

**Purpose:**

Autonomic dysfunction and a chronic low-grade inflammation are supposed to play a role in the etiology of major depressive disorder (MDD). The vagus nerves (VN) form a major part of the parasympathetic nervous system and of the gut-brain axis. They are supposed to exert anti-inflammatory and epithelial barrier protective effects in the gut. A reduced vagal activity was described in patients with MDD. We aimed to examine the VN in patients with MDD with high-resolution ultrasound (HRUS) and hypothesized that the cross-sectional area (CSA) and the echogenicity of the VNs were altered in comparison to healthy controls.

**Materials and methods:**

The echogenicity (gray scale mean) and the CSA of the cervical VNs at the level of the thyroid gland and both median nerves were examined with HRUS in 50 patients with MDD and 50 matched healthy controls.

**Results:**

The left VN-CSA was significantly larger in the MDD group compared to the control group (1.7 ± 0.4 mm^2^ versus 1.5 ± 0.4 mm^2^; *p* = 0.045). The CSA of the right VN and both median nerves (MN) were similar between groups. In MDD subgroup analyses, recurrent depressive disorders were the main contributing factor for the left VN-CSA enlargement. Echogenicity was not altered in the VN and MN between groups.

**Conclusion:**

The enlargement of the left VN-CSA in patients with MDD, and especially in these patients with recurrent depressive disorders, might turn out as a promising imaging biomarker. Longitudinal studies are warranted to examine whether the VNs-CSA change in the course of MDD.

## Introduction

As the vagus nerves (VNs) are of particular importance in psychiatric and neurological disorders, sonographic research has witnessed a growing interest (1–3). The VNs constitute a crucial part of the parasympathetic autonomic nervous system (ANS). The functional imbalance between reduced VN activity and the sympathetic system results in autonomic dysfunction, comprising symptoms like palpitations, impairment of sleep, appetite, and gastrointestinal functioning in neuropsychiatric disorders, e.g., major depressive disorder (MDD) (4). Currently, antidepressant treatments, such as vagus nerve stimulation (VNS), where the left VN is used as a target for electrical stimulation, emerged as an option in treatment-resistant depression (5). Meta-analyses also demonstrated a negative association between vagal activity and inflammatory markers (6). A lack of vagal inhibition of systemic inflammatory processes seems to play a key role in the low-grade inflammation pathogenesis approach of MDD (7). Moreover, the VNs form a central part of the gut-brain axis by linking the gut and abdominal organs with the central nervous system, thus, enabling a bidirectional communication (8). Disturbances of the microbiota and the gut-brain axis are also supposed to contribute to the etiology of depression and anxiety disorders (8, 9).

High-resolution ultrasound (HRUS) allows the reliable examinations of the VNs *in vivo* (10). Previous findings showed that sonomorphological VN alterations and autonomic function may correlate in healthy probands (11). Morphological alterations of the cervical VNs were described in different neurological disorders. An atrophy was found in patients with Parkinson’s Disease (PD) [e.g., (1, 12)], while an enlargement of the VN-CSA was associated with autonomic dysfunction in patients with Guillain-Barré-Strohl syndrome (13).

So far, although there is cumulating evidence for a relevant role of the VNs in the etiology of MDD, there are no morphological examinations of the VNs in these patients *in vivo*. Thus, we aimed to examine the VNs in patients with MDD with HRUS and hypothesized that the CSA and the echogenicity of the VNs were altered in comparison to healthy controls.

## Materials and methods

This study was performed according to the ethical standards laid down in the 1964 Declaration of Helsinki and its later amendment. It was approved by the local Ethics Committee of the Medical Faculty at the University of Leipzig (reference number 425/19-ek). All participants gave informed and written consent for participation in medical research.

Based on studies that examined the size of the VNs in predominantly neurodegenerative disorders where the differences in the CSA of the asymmetric VNs varied between 10% (in case of the right VN) and 20% (in case of the left VN) (1), we calculated that, using a two-tailed test, 53 patients with MDD had to be examined to detect a difference in the CSA of 15% with a power of 0.8. The entire cohort comprised 100 adult subjects (50 patients with MDD and 50 healthy controls) and was balanced according to sex and age (Table 1). Participants were recruited from 06/2020 to 09/2021 from the inpatient ward of the Department of Psychiatry and Psychotherapy, University of Leipzig Medical Center.

**Table 1:** Demographic data of patients with major depressive disorder (MDD) and healthy controls.

Characteristics	MDD group (*n* = 50)	Control group (*n* = 50)	*p-*value
Male (*n*)	21	21	1.00°
Female (*n*)	29	29	1.00°
Age in years (mean, ± SD, range)	45 ± 16 (21–80)	46 ± 21 (22–80)	0.972^#^
Height in cm (mean, ± SD, range)	172 ± 10 (146–20)	173 ± 10 (153–192)	0.890^+^
BMI (kg/m^2^; mean, ± SD, range)	26.6 ± 5.7 (17.1–46.9)	24.5 ± 3.6 (19.5–38.0)	0.049^#^
**Medical history of comorbidities**			
Cardiac arrythmia [*n* (%)]	5 (10%)	1 (2%)	0.092°
Diabetes mellitus [*n* (%)]	3 (6%)	1 (2%)	0.307°
**Questionnaires**			
BDI score (median, range)	24.5 (6–46)	4 (0–20)	0.001^#^
PHQ-15 score (median, range)	13 (1–22)	4 (0–20)	0.001^#^
Duration of actual depressive episode in weeks (mean, SD, range)	25.39 ± 18.74^*^	0	
	4–80^*^		

+ Student’s *t*-test; ^#^Mann–Whitney *U*-test; °Chi-square test; BMI, body mass index; BDI, Beck Depression Inventory; PHQ-15, Patient Health Questionnaire-15; SD, standard deviation. ^*^One extreme outlier of 400 weeks duration was excluded.

All patients had to fulfill the clinical criteria of depression (F32.1–F32.2 and F33.1–F33.3) as defined by the International Statistical Classification of Diseases and Related Health Problems, 10th Revision. Diagnoses were confirmed during the treatment by psychiatric consultants.

Exclusion criteria were a medical history of polyneuropathy, epilepsy, neurodegenerative disorders, use of illegal substances, any addictive diseases, any psychiatric diagnoses in the control group, organic or psychotic psychiatric comorbidities, any relevant anxiety and / or obsessive compulsive disorders in the MDD group, a history of head injury, or acute somatic diagnoses during the time of examination. All participants underwent a profound neurological examination to exclude persons with clinically apparent yet hitherto unknown polyneuropathy or parkinsonism.

At the time point of study participation, all patients in the MDD group were on antidepressants and had psychotherapy. We did not assess how long and how often patients had psychotherapy before their participation in this study nor which kind of psychotherapy they had in before. We also did not assess the history of antidepressant intake before study participation.

To evaluate the severity of depression at the time of participation, all participants completed the Beck Depression Inventory (BDI) and Patient Health Questionnaire-15 (PHQ-15) with focus on somatic symptoms.

**Figure 1:**
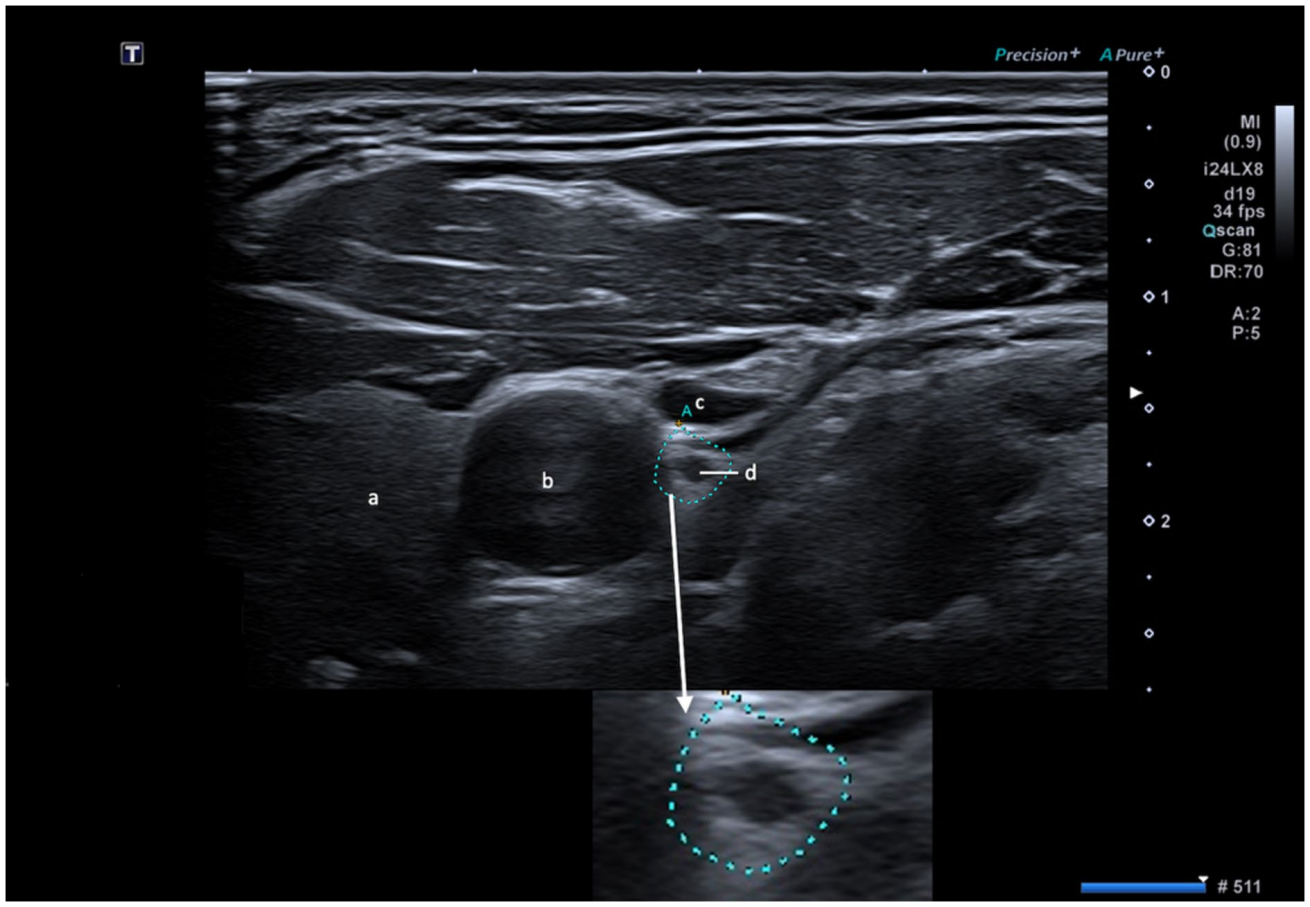
Visualization of the vagus nerve (d) with embedded magnification. a: thyroid gland; b: common carotid artery; c: internal jugular vein; scale bar = 1 cm.

The HRUS examination was performed with an Aplio i800 (Canon Medical Systems, Neuss, Germany) with a 24 MHz linear transducer. Briefly, both VNs, at the level of the thyroid gland, and, for control purpose, both median nerves (MN), 10 cm proximal to the wrist, were examined according to established protocols (10, 14) (Figure 1). Three B-mode images of each nerve and side were recorded and optimized regarding brightness, depth, and focus. The identified nerve was marked roughly with the marking tool of the ultrasound device, and the images were stored for *offline* measurement of the CSA. Post-examination *offline* measurements were performed with ImageJ (National Institutes of Health, Bethesda, Maryland, United States; version 1.53a). The CSA was determined with a precision of 0.1 mm^2^. Further statics were calculated with the median of the 3 CSA values of each nerve and side, which is less likely to be distorted by outliers.

In order to determine the echogenicity of the nerves, the image with the median CSA was converted into an 8-bit black and white image in which each pixel is assigned a grayscale. The grayscales ranged from 0 (black) to 255 (white). An average value of the grayscales of all pixels within the CSA was calculated (grayscale mean, GSM). To determine the echogenicity independently of brightness settings of the ultrasound device, the GSM of the VN was set in relation to the GSM of the blood in the ipsilateral common carotid artery (CCA), which is physiologically hypoechoic and shows little heterogeneity. The GSM ratio (GSM-VN / GSM-CCA) was used for further statistics.

All measurements were done by the same rater who was blinded to the side of the nerve (left vs. right) and to group affiliation (control vs. MDD).

Statistical analyses were performed by using IBM SPSS Statistics (IBM Corporation, Armonk, New York, United States; version 27.0). To assess intra-rater agreement 20 images were re-measured (ICC-coefficient = 0.996, *p* < 0.001). CSA values of one rater were used for statistical analyses. For group comparison, the student’s *t*-test (for data with normal distribution) and Mann–Whitney *U*-test (for non-normal distribution) were used. Chi-square test was applied on group comparisons of nominally scaled data. Correlation coefficients were calculated using Pearson’s correlation (normal distribution, metric level), Spearman’s correlation (non-normal distribution, ordinal level) and Eta Coefficient (nominal and metric level). The subgroup analysis was performed using Kruskal-Wallis one-way ANOVA. Extreme outliers were excluded based on Tukey’s hinges (first quartile −3 * interquartile range (IQR) and third quartile +3 * IQR), visualized in boxplots (15). The significance level was set at *p* < 0.05.

## Results

Demographic data of patients with MDD and the control group were well-balanced in terms of sex, age, and height. Only the BMI was significantly higher in the MDD group. No significant differences for known cardiac arrythmia or diabetes mellitus in the medical history were found between both groups. BDI and PHQ-15 scores were significantly higher in the MDD group. In the MDD group, the current depressive episode persisted at the time of examination for a mean of 25 weeks (after exclusion of one extreme outlier of 400 weeks; Table 1).

In HRUS examinations, the left VN-CSA was significantly larger in the MDD group than in the control group (*p* = 0.045), while the right VN-CSA did not differ significantly between groups (Table 2). In the MDD group, no significant correlations were found between the duration of the depressive episode and the left VN-CSA (ρ = −0.12; *p* = 0.413) or right VN-CSA (ρ = 0.04; *p* = 0.785), the BDI score and the left VN-CSA (ρ = −0.184; *p* = 0.201) or the right VN-CSA (ρ = 0.009; *p* = 0.952), nor the PHQ-15 score and the left VN-CSA (ρ = −0.134; *p* = 0.353) or the right VN-CSA (ρ = 0.031; *p* = 0.833). The left and right MN-CSA were similar between the control group and the MDD group (Table 2).

**Table 2:** High-resolution ultrasound data of patients with major depressive disorders (MDD) and healthy controls.

Variable		MDD group	Control group	*p-*value
VN-CSA left (mm^2^)	Mean, SD	1.7 ± 0.4	1.5 ± 0.4	0.045^+^
VN-CSA right (mm^2^)	Mean, SD	1.8 ± 0.5	1.7 ± 0.5	0.269^+^
MN-CSA left (mm^2^)	Mean, SD	6.9 ± 1.3	6.5 ± 1.2	0.079^+^
MN-CSA right (mm^2^)	Mean, SD	6.8 ± 1.3	6.5 ± 1.2	0.063^+^
VN GSM-Index left	Mean, SD	4.5 ± 2.4	5.3 ± 4.6	0.482^#^
VN GSM-Index right	Mean, SD	5.3 ± 4.7	6.1 ± 5.9	0.328^#^

+ Student’s *t*-test; ^#^Mann–Whitney *U*-test; HRUS, high-resolution ultrasound; VN, vagus nerve; CSA, cross-sectional area; GSM, gray scale mean; SD, standard deviation.

The MDD group was further stratified into two subgroups: first time diagnosis (FD; *N* = 18) and recurrent depressive disorder (RDD; *N* = 32; Table 3). Kruskal-Wallis one-way ANOVA revealed that the RDD subgroup contributed mainly to the significant enlargement of the left VN-CSA in comparison to the control group (*p* = 0.03; Table 4). For the right VN-CSA, Kruskal-Wallis one-way ANOVA showed no significant differences between MDD subgroups and the control group (Table 4).

**Table 3:** Demographic and high-resolution ultrasound data of subgroups of patients with major depressive disorder.

Characteristics	FD group (*n* = 18)	RDD group (*n* = 32)
Male (*n*)	7	14
Female (*n*)	11	18
Age in years (mean, ± SD, range)	39 ± 15 (22–64)	49 ± 17 (21–80)
Height in cm (mean, ± SD, range)	173 ± 11 (158–200)	172 ± 9 (146–191)
BMI (kg/m^2^; mean, ± SD, range)	24.2 ± 4.4 (17.1–31.8)	27.9 ± 5.9 (20.1–46.9)
**Questionnaires**		
BDI score (median, range)	27.5 (6–44)	23 (8–46)
PHQ-15 score (median, range)	13 (1–22)	12.5 (1–21)
Duration of actual depressive episode in weeks (mean, ± SD)	28.8 ± 21.2^*^	23.6 ± 17.4
**Ultrasound examination**		
VN-CSA left (mm^2^; mean, ± SD)	1.6 ± 0.4	1.7 ± 0.4
VN-CSA right (mm^2^; mean, ± SD)	1.9 ± 0.5	1.8 ± 0.5
MN-CSA left (mm^2^; mean, ± SD)	7.0 ± 1.6	6.9 ± 1.2
MN-CSA right (mm^2^; mean, ± SD)	6.9 ± 1.6	6.7 ± 1.2
VN GSM-Index left (mean, ± SD)	4.3 ± 2.4	4.6 ± 2.4
VN GSM-Index right (mean, ± SD)	5.7 ± 3.1	5.1 ± 5.4

FD, first time diagnosis; RDD, recurrent depressive disorder; SD, standard deviation; BDI, Beck Depression Inventory; PHQ-15, Patient Health Questionnaire-15; MN, median nerve; VN, vagus nerve; CSA, cross-sectional area; GSM, gray scale mean. ^*^One extreme outlier of 400 weeks duration was excluded.

**Table 4:** Subgroup analysis with Kruskal-Wallis one-way ANOVA between major depressive disorder patients with first time diagnosis (FD), recurrent depressive disorder (RDD), and control group for left and right vagus nerve (VN) cross-sectional area (CSA).

	Left VN-CSA	Right VN-CSA
	Adapted *p-*value	Adapted *p-*value
Control group/RDD	0.03	1.00
Control group/FD	1.00	0.683
FD/RDD	0.386	1.00

Regarding the echogenicity of the VNs, no significant differences were found between the control and the MDD group (Table 2), or its subgroups (Table 5). In both control and MDD group, a higher GSM-Index was measured for the right VN in comparison to the left VN (Mann–Whitney *U*-test *p* < 0.001; Table 2). In the MDD group, no significant correlation was found neither between the BDI score and the left (ρ = 0.143; *p* = 0.320) or the right GSM-Index (ρ = 0.201; *p* = 0.161), nor between PHQ-15 score and the left (ρ = −0.174; *p* = 0.277) or the right GSM-Index (ρ = 0.190; *p* = 0.186). In the whole study cohort, the right VN-CSA correlated significantly with the right GSM-Index (ρ = 0.227; *p =* 0.023), whereas the left VN-CSA and the left GSM-Index showed no significant correlation (ρ = −0.057; *p =* 0.571).

**Table 5:** Subgroup analysis with Kruskal-Wallis one-way ANOVA between major depressive disorder patients with first time diagnosis (FD), recurrent depressive disorder (RDD), and control group for left and right vagus nerve (VN) gray scale mean (GSM) index.

	Left VN-GSM-index	Right VN-GSM-index
	Adapted *p-*value	Adapted *p-*value
Control group/RDD	1.00	0.279
Control group/FD	1.00	1.00
FD/RDD	1.00	0.247

Sex, age, BMI, height, cardiac arrythmia, and diabetes mellitus were not identified as covariates for the VN-CSA nor for the VN echogenicity.

## Discussion

For the first time, this study revealed morphological changes of the cervical VNs in patients with MDD. The left VN-CSA was significantly enlarged in comparison to healthy subjects. Noteworthy, this enlargement of the left VN-CSA in patients with MDD was mainly driven by the subgroup of patients with recurrent depressive disorder.

Over the last decade, HRUS enabled the reliable examination of small nerves like the VN (10). A reduced VN-CSA was repeatedly measured in neurodegenerative disorders like PD or amyotrophic lateral sclerosis (1, 2, 12), while enlarged VN-CSA was described in inflammatory (13, 16, 17), but also in hereditary neuropathies (18). The enlarged left VN-CSA in patients with MDD and especially in the subgroup of patients with RDD might be due to a subtle inflammatory edema of the left VN. Other explanations like hereditary or inflammatory polyneuropathies (18) are unlikely because of the unaffected MNs, and participants with clinical signs of a polyneuropathy in the profound neurological examination were excluded from this study. Compression of nerves can also cause enlarged CSAs, however, during the HRUS examination the VN was visualized over its cervical course and no compression or entrapment was noted. Thus, the most probable explanation for the small (about 10%) but significant difference in the CSAs of the left VN remains a (chronic) inflammation which leads to an edema with subsequent VN enlargement. The VNs were also found to be enlarged in patients with chronic inflammatory demyelinating polyradiculoneuropathy (CIDP) (17, 19). Interestingly, patients with CIDP may also show a subtle affection of the ANS with focus on parasympathetic cardiovascular fibers (20), and they may suffer from neuropsychiatric symptoms and disorders like pain, fatigue, and depression (21, 22). However, so far depressive symptoms in patients with inflammatory polyneuropathies are thought to be reactive due to the patients’ functional impairment and not to be related to the inflammation of the peripheral nervous system or the VN (21).

The VNs with their afferent and efferent fibers also play a crucial role in connecting the gut and the brain. Recently, cumulate research suggested that a disturbance of the microbiota and the gut-brain axis might contribute to the etiology of depression (8, 9). In their review, Tan and colleagues argued that the immune response to gut microbiota translocation induced by a leaky gut may be responsible for the chronic inflammatory condition in depression. Pro-inflammatory cytokines like IL-2, IL-12, or TNF-α were repeatedly shown to be over-expressed in patients with MDD which points to a role of inflammation in the pathophysiology of MDD (23–28). The TNF-α inhibitor etanercept was effective in treatment-resistant depression and reduced depression and anxiety in psoriasis patients (29, 30). Consequently, modulating inflammation and immune regulation in patients with MDD emerged as a potential drug target (31).

Furthermore, the VNs may exert anti-inflammatory and epithelial barrier protective effects in the gut (8). The interactions between the immune system and the central nervous system are characterized by a bidirectional communication that aims to specify the immune defense of the host (32). Physiologically, the afferents of the VNs can sense a peripheral infection and transmit this information to the central nervous system which is shielded from the rest of the body by the blood brain barrier (32). This information may then be redirected to vagal efferents which can send anti-inflammatory responses through the inhibition of pro-inflammatory cytokines such as tumor necrosis factor (TNF)-α, interleukin (IL)-1, and the release of anti-inflammatory cytokines such as IL-10 (7, 33). This is also referred to as the “inflammatory reflex” of the VN (7, 33, 34). Thus, an (ongoing) inflammation of the VNs could restrict vagal activity and might lead to a lack of vagal downregulation of inflammatory processes. A reduced vagal activity was repeatedly described in patients with MDD (35, 36), and was mitigated after the onset of antidepressant treatment (36). The MDD subgroup analysis showed that the enlargement of the left VN-CSA was mainly driven by the RDD subgroup. We assume, that recurrent depressive episodes might trigger a chronification of vagal dysfunction by overstressing the anti-inflammatory functions of the VNs in the long term (37).

Considering side-specific effects of the VNs, in healthy subjects, Pelz et al. found an *inverse* correlation only between the left VN-CSA and parameters of parasympathetic activity (11). Left VN efferent neurons were also prominently involved in anti-inflammatory effects, at least in mice, where the selective stimulation of efferent cholinergic VN neurons originating in the left dorsal motor nucleus and projecting to the celiac-superior mesenteric ganglia significantly increased splenic nerve activity and inhibited TNF-α production (38). The so-called cholinergic anti-inflammatory pathway is exerted through vago-parasympathetic reflexes via the splenic nerve and vagal efferent neurons to enteric neurons resulting in a decrease of TNF-α (33). In humans, invasive VNS was approved for severe treatment-resistant depression in 2005 by the US Food and Drug Administration. Usually, the left cervical VN is stimulated (5, 39). Recently, left VNS also emerged as a promising treatment approach for inflammatory bowel disease (40, 41).

So far, echogenicity of nerves was examined only in a few studies. Gamber and colleagues did not find a general difference in the nerves’ echogenicity between patients with CIDP and probands, but differences between the subgroups of clinically progressive CIDP patients compared to healthy controls and stable CIDP patients (42). No differences were found in the echogenicity of the VNs between MDD and controls. One explanation may be, that the epineurium is relatively prominent, in particular in the right VN. Thus, a change in echogenicity was probably mitigated by the hyperechoic epineurium. However, there was a significant side difference of the GSM Index between the left and the right VN within both the MDD and control group. The GSM Index of the left VN was significantly lower than the right, i.e., the left VN was more hypoechoic, which could be a due to a lower number of fascicles in the left VN, which are sheathed by hyperechoic epineurium (10).

We found no significant correlation between MDD symptom severity and VN-CSA or echogenicity. BDI and PHQ-15 ask for symptoms within the last 2 weeks, which reflects rather acute than chronic symptoms. In our findings, the RDD subgroup contributed most to the alterations in VN-CSA. This may suggest that recurrent and chronic courses of MDD alters VN-CSA independently to its current symptom severity.

There are several limitations. Firstly, the MDD group was heterogenous, comprising patients with FD and RDD, with the RDD subgroup impacting the VN-CSA the most. Further HRUS investigations in MDD should focus on differences between first time, recurrent, and chronic depressive disorders. Moreover, it should be noted that the RDD subgroup presumably underwent a longer period of medical treatment, due to recurrent depressive episodes and long-term intake of antidepressants. We could not rule out that (especially the long-term-treatment with) antidepressants had an influence on the VN alterations, as they may also have anti-inflammatory effects (43, 44). Secondly, the left VN-CSA enlargement was small and thus, it appears unlikely that the VN-CSA may serve as a biomarker for diagnosis or treatment response in MDD on an individual basis. Thirdly, no general procedure of determining echogenicity in HRUS images is established yet. But unlike previous studies (42), we adjusted echogenicity for individual factors during the HRUS examination like changes in gain, depth, and focus by calculating an index, rather than reporting raw values. Finally, there is an ongoing debate whether the sonographically measured VN-CSA reflects the anatomical size of the VN (45).

In conclusion, the enlargement of the left VN-CSA in patients with MDD, and especially in these patients with recurrent depressive disorders, might turn out as a promising imaging biomarker. Possible mechanisms could involve a dysregulation of inflammatory and anti-inflammatory effects of the gut-brain axis. Further sonographic research is warranted, especially over the course of MDD to improve our understanding of the role of the VNs in affective disorders.

## Data availability statement

The raw data supporting the conclusions of this article will be made available by the authors, upon reasonable request.

## Ethics statement

The studies involving human participants were reviewed and approved by the Ethics Committee of the Medical Faculty at the University of Leipzig. The patients/participants provided their written informed consent to participate in this study.

## Author contributions

FS and JP designed the study. LS, DW, ES, and EB examined the participants. LS, JP, and FS analyzed the data. LS, DW, and JP wrote the manuscript. All authors contributed to the article and approved the submitted version.

## Conflict of interest

The authors declare that the research was conducted in the absence of any commercial or financial relationships that could be construed as a potential conflict of interest.

## Publisher’s note

All claims expressed in this article are solely those of the authors and do not necessarily represent those of their affiliated organizations, or those of the publisher, the editors and the reviewers. Any product that may be evaluated in this article, or claim that may be made by its manufacturer, is not guaranteed or endorsed by the publisher.
